# A cross-sectional study of acute dengue infection in paediatric clinics in Cameroon

**DOI:** 10.1186/s12889-019-7252-9

**Published:** 2019-07-18

**Authors:** Salomon Bonsi Tchuandom, Jules Colince Tchadji, Thibau Flaurant Tchouangueu, Monique Zambo Biloa, Etienne Philémon Atabonkeng, Marcelle Irina Miste Fumba, Eithel Sylvian Massom, Godwin Nchinda, Jules-Roger Kuiate

**Affiliations:** 1 Department of Biochemistry, University of Dschang, Dschang, Cameroon; 2 Laboratory of Vaccinology/Biobanking, CIRCB, Melen Yaoundé, Cameroon; 3 Public School of Medical Laboratory Technicians, Yaoundé, Cameroon; 4 Department of Animal Biology and Physiology, University of Yaoundé 1, Yaoundé, Cameroon; 5 Ministry of Public Health, Yaoundé, Cameroon; 6 FOSACO-IFSTMS, Yaoundé, Cameroon

**Keywords:** Fever, Acute dengue virus, Serologic markers, Children, Febrile illness, Cameroon

## Abstract

**Background:**

Dengue fever is the world’s fastest spreading mosquito borne viral infection. It is prevalent throughout both subtropical and tropical region, and affects over 128 countries. Dengue virus (DENV) infection poses a serious global public health challenge to three billion people, resulting in approximately 200 million cases of morbidity and 50,000 cases of mortality annually. In Cameroon like in most sub-Saharan African countries, DENV infection occur concurrently with other infectious diseases whose symptoms often overlap, rendering differential diagnosis challenging. This study aims at determining the frequency of acute dengue among febrile children under 15 years attending hospitals in some areas of Cameroon.

**Methods:**

A total of 961 children under the age of 15 were recruited in a cross-sectional study using systematic sampling technique and by selecting each subject out of the three. The study was conducted in 10 public health centers in Cameroon. Demographic data and risk factors of the subjects were obtained using well-structured questionnaires. Dengue virus NS1 antigen, IgM and IgG were analysed using a *Tell me fast®* Combo Dengue NS1-IgG/IgM Rapid Test. An in-house ELISA test for dengue specific IgM antibody was equally performed for confirmation. Descriptive statistical analysis was performed using Graph pad version 6.0.

**Results:**

A prevalence of 6.14% acute dengue virus infection was observed among children with febrile illness with a significant difference (*p* = 0.0488) between males (4.7%) and females (7.7%). In addition, children who reportedly were unprotected from vectors, showed a comparatively higher prevalence of the disease seropositivity than those practicing protective measures.

**Conclusion:**

DENV infection therefore is an important cause of fever among children in Cameroon. Thus, there is a need to include differential screening for DENV infections as a tool in the management of fever in children in the country.

**Electronic supplementary material:**

The online version of this article (10.1186/s12889-019-7252-9) contains supplementary material, which is available to authorized users.

## Background

Dengue, also known as “tropical flu,” is a viral disease transmitted to humans by *Aedes* mosquitoes [1]. This virus has four known circulating serotypes worldwide. A study carried out by Kamgang et al. (2017) [2] describes the presence of the *Aedes* species in some regions in Cameroon and others demonstrate that this vector bites only during the day [3–6]. Nearly three billion people live in high-risk areas, making dengue a significant public health problem. This infection is responsible for very high mobility and mortality in urban and suburban areas of high endemicity [7].

Since 2010, several African countries have suffered epidemic episodes of dengue including Tanzania, Zanzibar, the Comores, Benin, Cap Verde, Angola, Kenya, Somalia, Ivory Coast, Burkina Faso, Egypt and Nigeria [8, 9]. Sporadic DENV outbreaks have been reported in Nigeria since 1960 [10]. Cameroon shares many kilometers of borders with Nigeria and exchanges of goods and people has increasingly been growing in recent years [11]. The first identification of DENV in Cameroon was in 1987 [12], but several cases may remain undiagnosed, misdiagnosed or confused with other tropical diseases [7].

Classically, dengue occurs suddenly after 2 to 7 days of incubation with the onset of a high fever often accompanied by headache, nausea, vomiting, joint and muscle pain and a rash similar to that of measles. After 3 to 4 days, a brief remission is observed, and then the symptoms intensify before regressing rapidly after one week [13, 14]. In some cases, the disease can progress to severe forms: haemorrhagic dengue fever and dengue fever with shock syndrome that is fatal [13, 15, 16]. There is a need for early diagnosis of dengue infection since the early diagnosis of the disease can prevent fatal cases. The early diagnosis of dengue is based on the detection of the NS1 antigen, while the search for IgM antibodies can only be done after the 6th day after the onset of fever [17].

Confirmation of the diagnosis can be direct by detection of viral genomic DNA or its SN1 antigen, or indirect by detection of specific antibodies (IgM and IgG) in the blood. This may permit to distinguish acute primary and acute secondary dengue infections [18]. Early diagnosis can be obtained by gene amplification (RT-PCR) or by detection of NS1 antigen of the virus [18–21]. NS1 antigen is a glycoprotein protein detected from the first to the fifth day after the appearance of clinical signs by immunoenzymatic technique or immunochromatographic [18, 20, 22]. The viability and replication of the virus seems to depend on this protein [20, 23]. It stimulates a strong humoral response [24]. NS1 antigen is detectable in blood from first day after the onset of fever up to day 7, and still detectable in the presence of IgM antibodies, and when viral RNA is negative by RT-PCR [13, 21]. Simultaneous detection of NS1protein and IgM antibodies markers is useful to diagnose acute dengue infection [7, 25, 26]. This study therefore aims to determine the frequency of acute dengue infection among febrile children under 15 years attending hospitals in some selected areas of Cameroon.

## Methods

### Study and research design

This study was carried out in 10 health centers located in five regions of Cameroon as previously described [27].

### Description of study sites

The study was conducted in semi-urban areas (district hospitals of Kaelé (Far North), Bankim (Adamaoua) and Ntui (Centre)) and urban areas (district hospitals of Bafia (Centre), Edéa (Littoral), Yaoundé ‘Centre), Douala (Littoral), Bangangté, Foumban and Dschang (West)) [27].

### Inclusion criteria

Children with oral temperature ≥ 38 °C, fever < 7 days with at least one of specific symptoms (Head ache, joint pain, back ache, abdominal pain, vomiting, fatigue, anorexia and diarrhea), aged 4 months to 15 years recorded in the clinic regardless of gender, ethnicity or tribe, were included (Additonal file 1).

### Exclusion criteria

Children admitted in the hospital for known diseases or with fever of more than seven days and, above the age of 15 years were excluded.

### Sample size determination

The size of the population was determined using the following formula: n = Z^2^PQ/d^2^, where n is the sample size, P the prevalence, Q is 1-P, Z is the statistic level of confidence (1.96), d is precision (0.05) [28]. *P*-value was considered as 50%, representing maximum uncertainty [29]. Hence, the estimated sample size was 384.

### Sample collection and processing

Plasma was obtained after centrifugation of the blood (3 mL) of each patient, collected by venepuncture and introduced into EDTA tubes. Aliquots of each plasma were prepared in cryovial plain tubes and stored at − 20 °C. for subsequent analyzes.

### Laboratory test for dengue infection

Each plasma was analysed for the detection of NS1 antigen and for the detection of IgM antibodies using a *Tell me fast®* Combo Dengue NS1-IgG/IgM Rapid Test (Biocan Diagnostics Inc. Canada). The interpretation of the RDTs results was done in conformity with the manufacturer’s instructions. IgM and IgG antibodies against DENV were confirmed using an in-house indirect ELISA assay, as previously described [27, 30].

A confirmed acute dengue case was defined by the detection of the DENV NS1 protein alone or DENV NS1 protein with IgM antibodies or DENV NS1 protein with IgG antibodies against DENV in plasma samples of children with fever illness and at least one of the following symptoms: head ache, joint pain, back ache, abdominal pain, vomiting, fatigue, anorexia and diarrhea [26, 31, 32].

### Statistical analysis

The data from the operation of the questionnaire administered to the participants (spplement material) were processed statistically using the Graphpad Prism software. Seropositivity rates were caloked by considering acute dengue cases, characterized by the presence of NS1 antigen alone or associated with IgG / IgM. The Chi2 test was used to identify the associations between the different independent variables and their significance levels. Using bivariable logistic regression, we examined the effects of wearing long sleeve shirts and trousers, using indoor insecticide sprays and covering household water container on the odds of seropositivity were examined.

## Results

In this study, 961 febrile children were screened for seroprevalence of acute DENV using a *Tell me fast®* Combo Dengue NS1-IgG/IgM Rapid Test (Biocan Diagnostics Inc. Canada). As shown in Table 1, out of the 961 febrile children tested, 494 (51.4%) were males and 467 (48.6%) females, with a general age mean (± SD) 7.1 ± 2.9 years and age ranged from (min-max) of four months-15 years. Almost half of the subjects, 48.8% (469/961) were aged between one and five years, followed by those aged above five years, 43.8% (421/961). The children under one year old made up to 7.4% (71/961). A large portion, 61.4% (590/961) of febrile children included in the study lived in urban areas.

**Table 1:** Socio-demographic representation of the study population

Variables	Gender		Age groups		
	Females (n = 467)	Males (n = 494)	4 mth-1 yr (n = 71)	1–5 yrs (n = 469)	6–15 yrs (n = 421)
Semi urbain (%)	185 (39.6)	189 (38.3)	36 (50,7)	100 (21.3)	238 (56.5)
Urbain (%)	282 (60.4)	305 (61.7)	35 (49.3)	369 (78.7)	183 (43.5)

*mth*: months, *yr.(s)*year(s)

Noticeably, and as shown in Table 2, the febrile children who were seropositive for DENV-NS1 antigen (acute dengue: *n* = 59/961, 6.14%) were subdivided as follows: 5.1% (3/59) of children showed mono-positivity to DENV-NS1 alone; 84.7% (50/59) and 10.2% (6/59) of them showed dual positivity to DENV-NS1 plus anti-DENV IgM or IgG antibodies respectively. The subjects under one year were not positive to acute dengue marker (Table 2). However, children aged above one year predominantly expressed acute dengue. Nevertheless, acute dengue was not significantly associated with the age groups of children (*P* = 0.0815) (Table 2). The prevalence of acute dengue in females, 7.7% (36/467), was significantly higher (*P* = 0.0488) than in males, 4.7% (23/494). On the other hand, the prevalence of acute dengue in children living in urban areas, 5.9% (35/590) (Bafia, Edea, Yaoundé, Douala, Bangangté, Foumban and Dschang) was significantly not different (*p* = 0.7357) from that of the subjects living in semi-urban areas, 6.5% (24/371) (Table 2). Among the study sites, Ntui showed the highest frequency of dengue virus markers with 9.8% (7/71) of infections, while the subjects living in Foumban were less exposed, with just 4.0% (4/101) of dengue virus markers.

**Table 2:** Distribution of acute dengue, serological markers in febrile children according to different variables

				Dengue serological markers			
Variables			Children with acute dengue (%)	NS1 (%)	NS1 + IgM (%)	NS1 + IgG (%)	*P*-value (Chi-square)
Gender	Female (n = 467)		36 (7.7)	1(0.2)	34(7.3)	1(0.2)	0.0488* (3.88)
	Male (n = 494)		23 (4.7)	2(0.4)	16(3.2)	5(1.0)	
Age groups	4 mth-1 yr (n = 71)		0	0(0.0)	0(0.0)	0(0.0)	0.0815^**a**^ (5.015)
	1–5 yrs (n = 469)		31 (6.6)	3(0.6)	26(5.5)	2(0.4)	
	6–15 yrs (n = 421)		28 (6.6)	0(0.0)	24(5.7)	4(0.9)	
Residence	Semi-urban	Kaele (*n* = 150)	10(6.7)	1(0.7)	7(4.7)	2(1.3)	0.7357^**b**^ (0.1139)
		Bankim (n = 150)	7(4.7)	1(0.7)	5(3.3)	1(0.7)	
		Ntui (n = 71)	7(9.8)	0(0.0)	5(7.0)	2(2.8)	
	Urban	Bafia (*n* = 60)	4(6.7)	0(0.0)	3(5.0)	1(1.7)	
		Edea (*n* = 68)	4(5.9)	1(1.5)	3(4.4)	0(0.0)	
		Yaounde (*n* = 85)	8(9.4)	0(0.0)	8(9.4)	0(0.0)	
		Douala (*n* = 77)	4(5.2)	0(0.0)	4(5.2)	0(0.0)	
		Bangangte (*n* = 96)	5(5.2)	0(0.0)	5(5.2)	0(0.0)	
		Foumban (*n* = 101)	4(4.0)	0(0.0)	4(4.0)	0(0.0)	
		Dschang (*n* = 103)	6(5.8)	0(0.0)	6(5.8)	0(0.0)	

*mth* months, *yr.(s)* year(s), prevalence of acute Dengue with respect to the residence

Concerning the risk factors related to acute dengue in this study (Table 3), it was shown that 10.3% (52/503) of children who did not consistently cover their household water containers were positive for dengue virus markers, whereas those who cover their own were acute dengue positive at 1.5% (7/458). Furthermore, dengue virus markers were significantly (*p* = 0.0001) more prevalent among children who did not regularly wear long-sleeved shirts and pants (15.1%; 42/278) compared to those who did not wear them (2.5%; 17/683). The same trend has been observed among those who live in houses where insecticide is regularly sprayed (16.1%; 38/236), compared to those who live in houses where this practice is not common (2.9%; 21/725). There was also a statistical significance between the frequent wearing of long sleeve shirts with trousers and the presence of dengue virus markers (*p* = 0.0001). The level of Dengue virus markers was also relatively higher in patients who did not use indoor insecticide spray with a prevalence rate of 16.1% (38/236) compared to their counterparts who frequently used these insecticides sprays with a prevalence of 2.9% (21/725). Furthermore, there was a statistical difference between the frequent use of indoor insecticide sprays and the presence of dengue virus markers (p = 0.0001). Preventive behaviors such as the wearing of long sleeve shirts and trousers and the use of indoor insecticide spray obviously was associated with reduced risk of dengue infection in children (by 83.53 and 82.73%) by the mosquito bites with a relative risk (RR) of 0.1647 and 0.1727 respectively. Practices such as covering household water containers limited mosquito proliferation, and consequently reduced the risk of dengue infection in children with a relative risk of 0.1478, they were thus protected (85.22%).

**Table 3:** Evidence of dengue infection in febrile children according to risk factors

Risk factors	DENV Positive (%)	DENV Negative (%)	*p*-value	RR (95% CI)
Covered household water container			0.0001*	0.1478 (0.0678–0.32)
Yes (*n* = 458)	7 (1.5)	451 (98.5)		
No (*n* = 503)	52 (10.3)	451 (89.7)		
Frequency of wearing long sleeve shirts and trousers			0.0001*	0.1647 (0.0249–0.2844)
Yes (*n* = 683)	17 (2.5)	666 (97.5)		
No (*n* = 278)	42 (15.1)	236 (84.9)		
Frequency of indoor insecticide sprays			0.0001*	0.1727 (0.0702–0.2884)
Yes (*n* = 725)	21 (2.9)	704 (97.1)		
No (*n* = 236)	38 (16.1)	198 (83.9)		

## Discussion

The diagnosis of dengue requires either the direct detection of the virus or the detection of specific antibodies. While the use of molecular methods, including PRC, is not always easy to implement in developing countries, the ELISA method for the detection of NS1 antigen or specific IgG and IgM antibodies is easier to implement and at relatively low cost. The NS1 protein is an early marker of dengue infection and can be used to diagnose the disease in its acute phase [33, 34]. The use of this marker in this study allowed the prevalence of the acute dengue to be estimated at 6.14%; its combination with the detection of specific IgM increases the level of diagnostic sensitivity of acute dengue, which contributes to take rapid clinical decisions [35]. The prevalence obtained shows that dengue infection is responsible for a relatively large proportion of febrile states in children in Cameroon and that such diagnosis should be practiced even in rural areas. Fortunately, the Dengue NS1-IgG/IgM based RDTs Combo can be easily implemented in rural areas with good efficacy [36].

The prevalence value of acute dengue fever obtained in this study is very close to that reported by Nasir et al. (8.8%), in febrile patients at the University Hospital of Abuja (Nigeria) [37], but lower than those of 35% reported in Ibadan [38], 77.1% in Nnewi [39] and 30.8% in Kwara state in Nigeria [40]. Thus, the differences observed could result from the fact that, unlike the present study, some of these authors have relied on the detection of dengue-specific IgM to identify acute dengue cases, which in the absence of the NS1 antigen may refer only to a secondary dengue and thus a late diagnosis [13, 41]. Nevertheless, false positive results of NS1 antigen detection have been reported for this dengue protéin in a patient with acute Zika virus infection [42] while regions of cross-reactivity with Zika virus have not yet been tested. For this reason, it is better to combined dengue-specific antigen (NS1) and specific antibodies (IgM and IgG) to increase the diagnosis rates for dengue [23, 43, 44]. This study showed that children under one year of age were less affected by dengue fever while 6.6% of older children were dengue positive, consistent with earlier findings in Nigeria [45]. The explanation for this trend lies in the fact that the mosquito vector of dengue mainly stings out of the houses. For example, one-year-old children, in addition to being better protected, are rarely found outside homes where they are more likely to come into contact with mosquitoes. The prevalence of acute dengue fever was significantly higher in women than in men, which is consistent with recent studies in Indonesia and Thailand [38, 46–48], but contrasts with another study in India [49].

In the present state of knowledge, no studies on hospital seroprevalence of acute Dengue have been reported in Cameroon. In this study, a relative higher prevalence of acute dengue was observed in children who did not adopt various protective measures against the mosquito bites, compared to those who practiced some protective measures such as frequency of wearing long sleeve shirts and trousers as well as frequency of indoor insecticide sprays. These observations may are in line with previous studies carried out in Pakistan [49–51] but contrast with those of Mustapha et al. [52] who realized no association between dengue virus infection and protective measures.

Finally, we would have probably found more acute dengue among febrile children in Cameroon if children with febrile illness in all health centers were included in the study. Dengue RT-PCR serotype and molecular characterization strains should be used in the study environment.

## Conclusion

Dengue is a neglected disease and. This study has reported seroprevalence of acute dengue among febrile children in Cameroon for the first time. The low seroprevalence of acute dengue in the study population may be an indication of potential endemicity of the infection in the general population of the country. The results of this study show that dengue fever is potentially endemic in Cameroon, suggesting that appropriate measures should be taken by public health officials to limit children’s exposure to risk factors. In addition, we suggest that the diagnosis of dengue be included in routine examinations of febrile children in Cameroon hospitals to avoid the occurrence of epidemics in the population. Lastly, a special attention should be paid to the epidemiological surveillance of this viral infection in Cameroon.

## Additional file

Additional file 1: Fact Sheet for participant data collection. (DOCX 15 kb)

## Data Availability

The datasets used and/or analyzed during the current study are available from the corresponding author on reasonable request.

## Abbreviations

DENV: Dengue virus
DHF: Dengue haemorrhagic fever
DSS: Dengue shock syndrome
ELISA: Enzyme linked Immunosorbent assay
IgG: Immunoglobulin G
IgM: Immunoglobulin M
NS1Ag: Non-structural antigen
RNA: Ribonucleic acid
RT-PCR: Reverse transcriptase polymerase chain reaction
