# Supplementary material for: A cross-sectional study of acute dengue infection in paediatric clinics in Cameroon
Source: BMC Public Health. 2019 Jul 18;19:958. doi: 10.1186/s12889-019-7252-9 (PMC6637490; doi:10.1186/s12889-019-7252-9)
Supplement: Supplementary file 1 — Fact Sheet for participant data collection. (DOCX 15 kb) [file 12889_2019_7252_MOESM1_ESM.docx]

**Annex 2.6: Fact Sheet / Questionnaire**

**CODE : /___________________________/**

| *INFORMATION !* |
| --- |
| The information of the participants will be reconciled in this questionnaire; this information is confidential and reserved for members of the research team and the participant. Their exploitation will contribute to research on the distribution of dengue in Cameroon. |

1. **Identification**

Date of birth : /___________ / Region of origin: /____________________/

Residence : /_________________________/ Have you stayed anywhere else in the last 2 weeks? yes /___/ no /___/

If yes, which region? /__________________________/ for how long? (week) /_________/

Phone number : /_________________________/

How many people live in your household: /________/

Sex : Male /__/ Female/___/

If Female, pregnant ? : yes /__/ no /__/ If yes, week of amenorrhea /______/

Malaria treatment yes /___/ no /___/

1. **Use of mosquito net**

Do you use mosquito nets on top of your bed? yes/____/ no /____/ Simple : yes /____/ no /____/ impregnated: yes /____/ no /____/

**Environment**

**Exposure to water**

In the list below, which water materials are found in your house or in the neighborhood?

Tap for water : yes / ___ / no / ___ /,

Water tanks : yes / ____ / no ____

Flower boxes yes/ ____ / no ____,

can : yes / ____no ____ /,

Old abandon tires : yes / ____no____ /

abandon car bodies : yes/ ____ no _____/,

abandon pan : yes/ ___ no ____/ .

Do you stock water in your house? yes / ___ / no / ___ / If yes, is the container covered? Yes/____/ No /___/

Do you wear long sleeves? Yes/_____/ No /___/

1. **Clinical parameters**

Reason (s) for consultation / _____________________________________________________________________ /

Temperature? / _____ /

Blood pressure? / ____ / _____ /

Fever? yes / __ / no / __ / If yes, how many days? / ____ / Chills? yes / ___ /no / ___ /

Headache? yes / __ / no / __ /

Muscle pain? yes / __ / no / __ /

Lumbar pain? yes / __ / no / __ /

General Fatigue? yes / __ / no / __ /

Joint pain? yes / __ / no / __ /

Retro-Orbital pain? yes / __ / no / __ /

Headache? yes / __ / no / __ /

Cough? yes / __ / no / __ /

Gastrointestinal disorders? yes / __ / no / __ /

Hemorrhages? yes / __ / no / __ /

Tasks of redness on the skin? yes / __ / no / __ /

Hospitalization? yes / ___ / no / ___ / If yes, how many days / _______ /

1. **Biological parameters**

Full blood count prescribed? yes / __ / no / __ /

Liver function tests: yes / ___ / no / ___ /

Renal function tests: yes / ___ / no / ___ /

CRP: positive / ___ / negative / ___ /

Malaria parasite: positive / ___ / negative / ___ /

Typhoid fever: positive / __ / negative / ___ /

Erythrocyt sedimentation rate: positive / ___ / negative / ___ /

Hemostasis tests (: positive / ___ / negative / ___ /

**Thank for your participation**
